# Pregnant women’s perceived understanding and antenatal information needs regarding newborn screening for inherited metabolic diseases in southwestern China: a cross-sectional study

**DOI:** 10.3389/fpubh.2026.1865107

**Published:** 2026-10-06

**Authors:** Hao Yang, Fenghua He, Dan Xia, Xihong Wang, Hongqian Liu

**Affiliations:** 1 Center for Medical Genetics, West China Second University Hospital, Sichuan University, Chengdu, China; 2 Key Laboratory of Birth Defects and Related Diseases of Women and Children (Sichuan University), Ministry of Education, Chengdu, China; 3 West China School of Public Health and West China Fourth Hospital, Sichuan University, Chengdu, China

**Keywords:** antenatal information engagement, attitudes, China, inherited metabolic diseases, newborn screening, perceived understanding, pregnant women, screening and follow-up intentions

## Abstract

**Background:**

Newborn screening (NBS) can reduce morbidity from some inherited metabolic diseases (IMDs) through early detection and timely treatment. Clear antenatal communication can support informed participation and timely follow-up by helping parents understand the purpose and pathway of screening. Evidence on pregnant women’s perceived understanding, attitudes, antenatal information engagement, and screening- and follow-up-related intentions regarding newborn IMD screening in China remains limited. This study described these four domains and examined their associations with sociodemographic and reproductive-history characteristics in southwestern China.

**Methods:**

This hospital-based cross-sectional survey used a purposive sample recruited at a tertiary women’s and children’s hospital (National Regional Medical Center in China) from January 2024 to December 2024. After data-quality review, 1,320 eligible records were analyzed. All 21 questionnaire items were retained and analyzed as four domains: perceived understanding, attitudes, antenatal information engagement, and screening/follow-up intentions. Internal consistency, *post hoc* exploratory factor analysis, Pearson correlations, and four fully adjusted linear models with HC3 robust standard errors were evaluated. Benjamini-Hochberg false-discovery-rate (FDR) correction was applied across 92 non-intercept coefficients.

**Results:**

Parallel analysis supported four factors (Kaiser-Meyer-Olkin = 0.912; Bartlett χ^2^ = 21,719.21, *p* < 0.001), explaining 67.5% of variance. Mean (SD) scores were 15.15 (4.54)/35 for perceived understanding, 25.43 (3.77)/30 for attitudes, 9.34 (2.31)/15 for antenatal information engagement, and 20.09 (3.16)/25 for intentions; Cronbach’s α values were 0.918, 0.950, 0.745, and 0.914. Fifteen coefficients had nominal *p* < 0.05, but none remained significant after FDR correction (all q ≥ 0.051); adjusted R^2^ values were 0.010–0.039.

**Conclusion:**

Participants reported limited self-perceived understanding alongside favorable attitudes, moderate antenatal information engagement, and strong stated intentions. Adjusted R^2^ values of 0.010–0.039 and the absence of FDR-significant predictors indicate that the measured sociodemographic and reproductive-history variables had negligible explanatory value and should not be used for subgroup prediction or targeting. The domain pattern supports universal, nursing-led antenatal communication focused on screening procedures, abnormal-result interpretation, recall, confirmation, and follow-up, while future work should refine the questionnaire and test whether education improves observed participation and follow-up.

## 1 Introduction

Inherited metabolic diseases (IMDs) are heterogeneous genetic disorders that disturb metabolic pathways and may cause toxic-metabolite accumulation or deficiency of essential products (1, 2). Some screened conditions are clinically silent at birth but can lead to irreversible neurological injury, disability, or death if diagnosis and effective treatment are delayed (2, 3). NBS is therefore a population-based public-health system linking presymptomatic testing with recall, diagnostic confirmation, treatment, and longitudinal follow-up (3, 4). Screening panels and program design vary substantially across jurisdictions (4, 5). Education cannot prevent the underlying pathogenic variant; its defensible role is to support informed participation and timely access to screening, confirmation, and treatment.

In China, national policy promotes screening for phenylketonuria and congenital hypothyroidism, alongside hearing-loss and congenital-heart-disease screening, and supports gradual expansion where capacity permits (6, 7). The 2023–2027 national plan sets a 98% target for neonatal IMD screening and emphasizes recall, diagnosis, treatment, intervention, and follow-up (6). Nationwide MS/MS activity expanded from 2017 to 2021, but regional screening strategies and support systems remained heterogeneous (8). Recent Chinese studies further demonstrate regional disease spectra, substantial recall workloads, cost-effectiveness considerations, and the emerging complementary role of genomic screening (9–12). Therefore, routine program organization does not eliminate the relevance of family communication, optional expanded panels, local financing, or response to an abnormal result.

The 2025 ISNS guideline treats the screening offer, information, sample collection, laboratory testing, referral, confirmatory diagnosis, treatment, and follow-up as a connected pathway and recommends culturally appropriate information, ideally during pregnancy (13). NBS-specific studies show that antenatal information can be limited and that pregnant women or parents may support screening while lacking procedural knowledge (14–17). False-positive results can cause family distress (18, 19), and families of children with an IMD identified by NBS may experience psychosocial and financial burden (20). These findings support preparation for consent, recall, confirmation, and follow-up without implying that maternal perceptions create the screening program or determine coverage.

## 2 Methods

### 2.1 Study design and setting

This hospital-based cross-sectional survey was conducted at a tertiary women’s and children’s hospital (a National Regional Medical Center in China) in southwestern China from January to December 2024. Participants were recruited by purposive sampling. Reporting follows the STROBE recommendations (21).

### 2.2 Participants, sample size, and analytic sample

Eligible participants were pregnant women with a confirmed pregnancy who could read and complete the questionnaire independently and provided electronic informed consent. Repeat submissions and responses that could not be completed because of communication barriers were excluded. The minimum sample size was calculated using n = Z^2^p (1 − p)/d^2^ with Z = 1.96, *p* = 0.50, and d = 0.05 (22). Adding 10% to the minimum required sample size gave a target of 423. The source record documented 1,409 returned questionnaires, of which 89 invalid, incomplete, internally inconsistent, or implausibly rapid submissions were excluded. Therefore, all 1,320 eligible participants (93.7% of returned questionnaires) were included in the analytic sample.

### 2.3 Data collection and confidentiality

Data were collected through an online questionnaire after electronic informed consent. The platform recorded names and telephone numbers for follow-up; the survey was therefore confidential rather than anonymous. Direct identifiers were excluded from all analyses and are not reported. All 21 item responses were complete in the final analytic sample.

### 2.4 Questionnaire development and construct definitions

The questionnaire contained demographic variables and 21 self-report items scored from 1 to 5. It was developed with reference to prior literature and reviewed by two experts, one in pediatric medicine and one in health education, each with more than 10 years of experience. No formal pilot study, cognitive interview, independent content-validity assessment, test–retest study, criterion validation, or external psychometric validation was conducted. The original item wording was not changed after data collection; any revised labels are analytic interpretations rather than retroactive questionnaire edits. Q1-Q7 were labeled perceived understanding (1 = very unclear; 5 = very clear). Q8-Q13 formed attitudes; Q14-Q16 formed antenatal information engagement; and Q17-Q21 were labeled screening- and follow-up-related intentions because they were declarations or hypothetical statements made before birth rather than observed practice. Exact wording and descriptive results for all 21 items are shown in Supplementary Table S1. Q2 and Q3 broadly concerned “common types” and “common symptoms” without delimiting the locally screened panel, while Q14 combined seeking and reading information but did not assess time, access, source credibility, or other barriers. These features were not modified retrospectively and are treated as measurement limitations. Domain scores summed Q1-Q7 (range 7–35), Q8-Q13 (6–30), Q14-Q16 (3–15), and Q17-Q21 (5–25). Higher scores indicate greater self-perceived understanding, more favorable attitudes, more reported antenatal information engagement, or stronger stated intentions. The four domains were analyzed separately because they represent conceptually distinct constructs.

### 2.5 Statistical analysis

Categorical variables are summarized as n (%), items and domains as mean (SD), and item response categories as percentages. Internal consistency was estimated with Cronbach’s α. The 21-item structure was examined *post hoc* using principal-axis exploratory factor analysis with varimax rotation. Sampling adequacy was assessed with the Kaiser-Meyer-Olkin statistic and Bartlett’s test. The number of factors was determined by Horn’s parallel analysis using 500 random datasets and a fixed seed (23); loadings ≥0.40 were considered salient. Pearson correlations with Fisher-z 95% confidence intervals described associations among domain scores. Fully adjusted ordinary least-squares models were fitted for each domain. Age, residence, education, household income, ethnicity, commercial medical insurance, family history of genetic disease, miscarriage history, diagnosed genetic disease, diagnosed genetic variant, diagnosed chromosomal abnormality, previous birth of a child with an IMD, and reported fetal abnormality in the current pregnancy were entered simultaneously. Multicategory variables were dummy-coded with explicit reference groups. HC3 heteroskedasticity-consistent standard errors and 95% confidence intervals were reported (24); the maximum variance-inflation factor was 3.34. Benjamini-Hochberg FDR-adjusted q values were calculated jointly across the 92 non-intercept coefficients (25). Two-sided nominal *p* < 0.05 and FDR q < 0.05 are shown, but interpretation emphasizes direction, confidence intervals, adjusted R^2^, and multiplicity-adjusted results. No imputation was required.

### 2.6 Ethics statement

Ethical approval was obtained from the Ethics Committee of West China Second University Hospital, Sichuan University ([2023] Lun No. (104)). Electronic informed consent was obtained from all participants. All procedures were conducted in accordance with institutional requirements and the Declaration of Helsinki.

## 3 Results

### 3.1 Participant flow and characteristics

The final analysis included 1,320 participants. Most were aged 35 years or younger (936/1,320, 70.9%), lived in urban areas (1,000/1,320, 75.8%), and had an associate degree or higher (983/1,320, 74.5%). Twenty-four participants (1.8%) reported a previous child with an IMD. Table 1 provides the main recorded characteristics.

**Table 1:** Participant characteristics (*n* = 1,320).

Characteristic	Category	*n*	%
Age	18–25	126	9.5
	26–30	373	28.3
	31–35	437	33.1
	36–40	328	24.8
	≥41	56	4.2
Residence	Urban	1,000	75.8
	Town	213	16.1
	Rural	107	8.1
Education level	High school or below	337	25.5
	Associate degree	396	30.0
	Bachelor’s degree	480	36.4
	Master’s degree or above	107	8.1
Monthly household income (CNY)	≤4,000	150	11.4
	4,001-8,000	411	31.1
	8,001-12,000	312	23.6
	12,001-16,000	163	12.3
	16,001-20,000	129	9.8
	≥20,001	155	11.7
Ethnicity	Han	1,263	95.7
	Minority	57	4.3
Commercial medical insurance	Yes	533	40.4
	No	787	59.6
Family history of genetic disease	Yes	61	4.6
	No	1,259	95.4
History of miscarriage	Yes	227	17.2
	No	1,093	82.8
Diagnosed genetic disease	Yes	42	3.2
	No	1,278	96.8
Diagnosed genetic variant	Yes	130	9.8
	No	1,190	90.2
Diagnosed chromosomal abnormality	Yes	82	6.2
	No	1,238	93.8
Previous birth of a child with an IMD	Yes	24	1.8
	No	1,296	98.2

### 3.2 Internal structure and domain distributions

The KMO statistic was 0.912 and Bartlett’s test was significant (χ^2^ = 21,719.21, *p* < 0.001). Parallel analysis retained four factors. The rotated solution explained 67.5% of item variance and was compatible with the four content-based domains (Table 2). Primary loadings ranged from 0.702 to 0.854 for perceived understanding, 0.764 to 0.885 for attitudes, 0.461 to 0.810 for antenatal information engagement, and 0.588 to 0.830 for intentions. Q14 had the weakest primary loading (0.461). These findings do not resolve the wording limitations. Mean (SD) domain scores were 15.15 (4.54) for perceived understanding, 25.43 (3.77) for attitudes, 9.34 (2.31) for antenatal information engagement, and 20.09 (3.16) for screening and follow-up intentions (Table 3). Cronbach’s α values were 0.918, 0.950, 0.745, and 0.914, respectively. Treatment methods (Q5) had the lowest perceived-understanding mean (1.86), and participation in IMD-related health education (Q15) had the lowest engagement mean (2.69). Figure 1 displays all item response distributions.

**Table 2:** Rotated principal-axis factor loadings for the all-item-retention structure.

Item	Abbreviated content	Perceived understanding	Attitudes	Antenatal engagement	Screening/follow-up intentions
Q1	Basic concepts	0.761	-	-	-
Q2	Common types	0.822	-	-	-
Q3	Common symptoms	0.854	-	-	-
Q4	Diagnostic methods	0.751	-	-	-
Q5	Treatment methods	0.702	-	-	-
Q6	Preventive measures	0.782	-	-	-
Q7	Risk factors	0.783	-	-	-
Q8	Importance for prevention/early treatment	-	0.828	-	-
Q9	Genetic counseling and prevention	-	0.832	-	-
Q10	Willingness for health education	-	0.764	-	-
Q11	Necessity of regular screening	-	0.869	-	-
Q12	Attitude toward treatment	-	0.885	-	-
Q13	Responsibility to raise awareness	-	0.867	-	-
Q14	Seeks and reads information	-	-	0.461	-
Q15	Participated in health education	-	-	0.810	-
Q16	Learning/prevention in pregnancy	-	-	0.723	-
Q17	Regular screening statement	-	-	-	0.588
Q18	Seeks professional advice	-	-	-	0.800
Q19	Shares information	-	-	-	0.814
Q20	Supports others’ screening/treatment	-	-	-	0.830
Q21	Treatment/care adherence statement	-	-	-	0.740

Varimax rotation; loadings with absolute value <0.40 are suppressed. KMO = 0.912; Bartlett χ^2^ = 21,719.21, *p* < 0.001; four factors retained by parallel analysis; variance explained = 67.5%. The solution is post hoc internal evidence, not external validation.

**Table 3:** Revised domain scores and internal consistency (*n* = 1,320).

Domain	Items	Possible range	Observed range	Mean (SD)	% of maximum	Cronbach’s α
Perceived understanding	Q1, Q2, Q3, Q4, Q5, Q6, Q7	7–35	7–27	15.15 (4.54)	43.3	0.918
Attitudes	Q8, Q9, Q10, Q11, Q12, Q13	6–30	6–30	25.43 (3.77)	84.8	0.950
Antenatal information engagement	Q14, Q15, Q16	3–15	3–15	9.34 (2.31)	62.3	0.745
Screening and follow-up intentions	Q17, Q18, Q19, Q20, Q21	5–25	5–25	20.09 (3.16)	80.4	0.914

**Figure 1:**
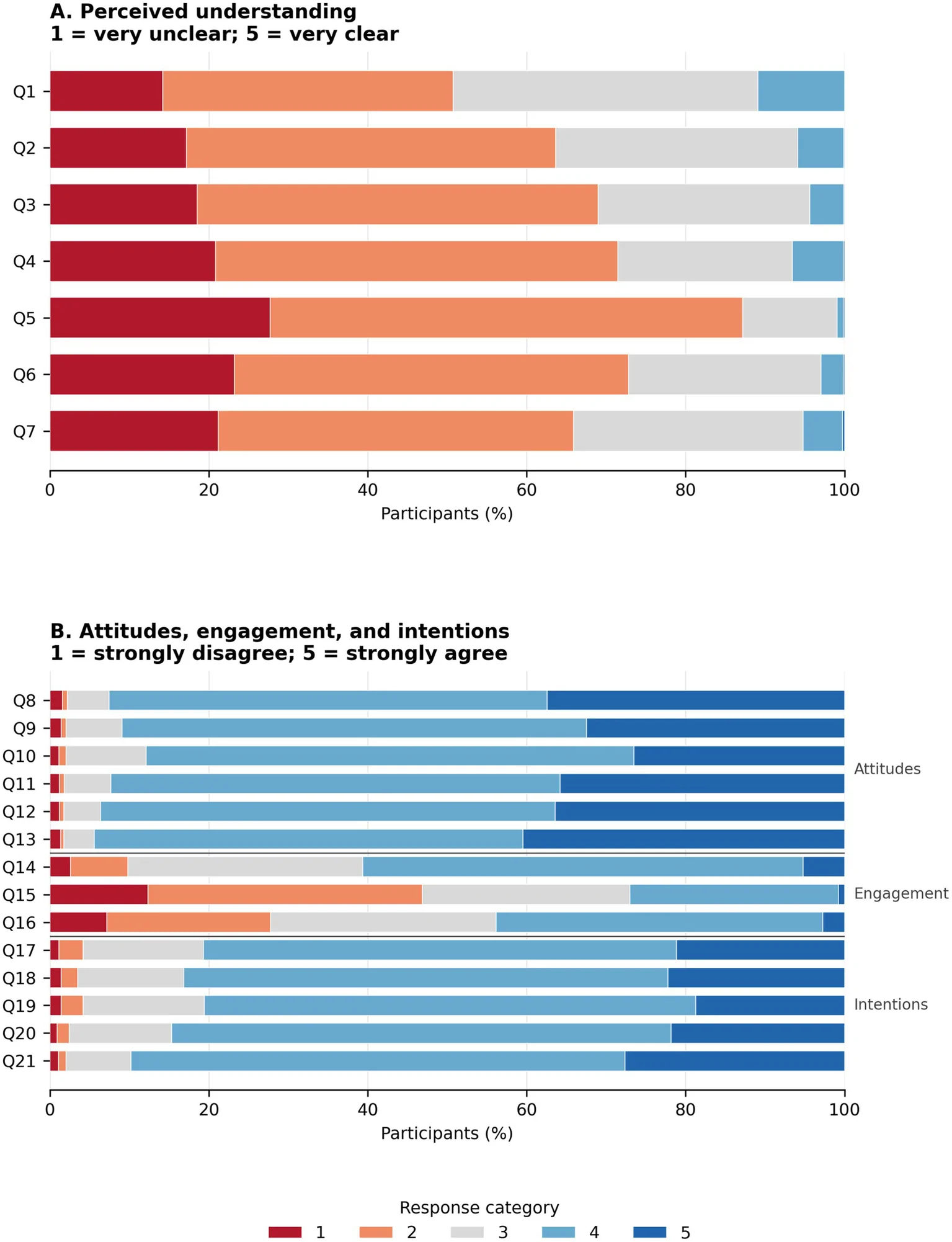
Item-level response distributions for all 21 items (*n* = 1,320). **(A)** Uses 1 = very unclear and 5 = very clear. **(B)** Uses 1 = strongly disagree and 5 = strongly agree. Exact wording and domain allocation are provided in Supplementary Table S1. Percentages may not sum to exactly 100 because of rounding.

### 3.3 Correlations among revised domains

Perceived understanding was not correlated with attitudes (*r* = 0.007, 95% CI − 0.047 to 0.061) and showed a very weak, non-significant correlation with intentions (*r* = 0.053, −0.001 to 0.106). It correlated moderately with antenatal information engagement (*r* = 0.353, 0.305 to 0.399). Attitudes correlated weakly with engagement (*r* = 0.135, 0.082 to 0.188) and strongly with intentions (*r* = 0.594, 0.558 to 0.628); engagement correlated weakly with intentions (*r* = 0.264, 0.213 to 0.313). These cross-sectional correlations do not establish temporal or causal pathways (Table 4).

**Table 4:** Pearson correlations among revised domain scores.

Domain	Compared domain	*r*	95% CI	*p*
Perceived understanding	Attitudes	0.007	−0.047 to 0.061	0.795
	Antenatal information engagement	0.353	0.305 to 0.399	<0.001
	Screening and follow-up intentions	0.053	−0.001 to 0.106	0.056
Attitudes	Antenatal information engagement	0.135	0.082 to 0.188	<0.001
	Screening and follow-up intentions	0.594	0.558 to 0.628	<0.001
Antenatal information engagement	Screening and follow-up intentions	0.264	0.213 to 0.313	<0.001

### 3.4 Exploratory multivariable associations

Fifteen contrasts had nominal *p* < 0.05 (Table 5), including lower perceived understanding for age 26–30 and 31–35 years than 18–25 years, higher perceived understanding for town than urban residence, higher attitudes for a bachelor’s degree than high school or below, and lower antenatal engagement for bachelor’s and master’s-level education. However, none remained significant after FDR correction (all q ≥ 0.051). Model R^2^ values were 0.027, 0.051, 0.055, and 0.027 for perceived understanding, attitudes, engagement, and intentions; adjusted R^2^ values were 0.010–0.039. Thus, after adjustment, the models explained only 1.0–3.9% of score variance. The nominal contrasts therefore have negligible explanatory and predictive value and do not justify causal inference, individual prediction, demographic targeting, or a conclusion that subgroup inequities are absent.

**Table 5:** Adjusted associations with nominal *p* < 0.05.

Outcome	Contrast	*B*	95% CI	*p*	FDR q	Direction	Adjusted R^2^
Perceived understanding	Age 26–30 (vs 18–25)	−1.313	−2.165 to −0.461	0.003	0.054	Lower	0.010
	Age 31–35 (vs 18–25)	−1.051	−1.929 to −0.172	0.019	0.160	Lower	0.010
	Town (vs urban)	1.123	0.433 to 1.814	0.001	0.051	Higher	0.010
	Income 4,001–8,000 (vs ≤ 4,000 CNY)	0.851	0.016 to 1.686	0.046	0.272	Higher	0.010
	Income 12,001–16,000 (vs ≤ 4,000 CNY)	0.936	0.005 to 1.868	0.049	0.272	Higher	0.010
	Income 16,001–20,000 (vs ≤ 4,000 CNY)	1.257	0.184 to 2.331	0.022	0.167	Higher	0.010
	Previous child with IMD: yes	2.568	0.619 to 4.517	0.010	0.113	Higher	0.010
Attitudes	Rural (vs urban)	−1.105	−1.916 to −0.294	0.008	0.113	Lower	0.034
	Bachelor’s degree (vs high school or below)	0.891	0.304 to 1.477	0.003	0.054	Higher	0.034
Antenatal information engagement	Bachelor’s degree (vs high school or below)	−0.604	−0.975 to −0.232	0.001	0.051	Lower	0.039
	Master’s degree or above (vs high school or below)	−0.950	−1.541 to −0.359	0.002	0.051	Lower	0.039
	Income 12,001–16,000 (vs ≤ 4,000 CNY)	−0.715	−1.268 to −0.162	0.011	0.116	Lower	0.039
	Income 16,001–20,000 (vs ≤ 4,000 CNY)	−0.761	−1.373 to −0.149	0.015	0.136	Lower	0.039
	Income ≥20,001 (vs ≤ 4,000 CNY)	−0.784	−1.377 to −0.190	0.010	0.113	Lower	0.039
Screening and follow-up intentions	Previous child with IMD: yes	1.499	0.054 to 2.944	0.042	0.272	Higher	0.010

Every model simultaneously included all prespecified predictors. Age 18–25 years, urban residence, high school or below, monthly household income ≤4,000 CNY, Han ethnicity, and “no” are reference groups. HC3 robust standard errors were used. FDR q values were calculated jointly across all non-intercept coefficients. Full coefficients are provided in Supplementary Table S2.

## 4 Discussion

### 4.1 Principal findings

Four findings warrant emphasis. First, participants reported relatively limited perceived understanding of newborn inherited metabolic diseases: the mean score was 15.15 of 35 (43.3% of the maximum), and treatment methods received the lowest item rating. Second, attitudes toward screening, health education, treatment, and shared responsibility were strongly favorable (25.43 of 30; 84.8% of the maximum). Third, antenatal information engagement was moderate and uneven (9.34 of 15; 62.3% of the maximum), with participation in relevant health-education activities lower than several other engagement behaviors. Fourth, screening- and follow-up-related intentions were strong (20.09 of 25; 80.4% of the maximum). Together, these findings describe a population that is broadly supportive of newborn screening but would benefit from more accessible and clinically focused antenatal information.

The four-factor solution and correlation pattern add meaning to the descriptive results. Perceived understanding was moderately associated with antenatal information engagement (*r* = 0.353), whereas attitudes were strongly associated with intentions (*r* = 0.594). By contrast, perceived understanding was essentially unrelated to attitudes (*r* = 0.007). These are domain-specific, cross-sectional associations rather than evidence of two causal pathways. The near-zero correlation is consistent with an attitude-understanding gap in this sample, but it does not establish whether that gap is universal or generated by local questionnaire wording, screening-program organization, or communication context. The findings therefore favor separate interpretation of the four domains and identify distinct targets for maternal health education.

### 4.2 Perceived understanding and antenatal information engagement

The combination of limited perceived understanding and only moderate information engagement is consistent with newborn-screening research showing that pregnant women and parents may strongly support screening while remaining uncertain about screened conditions, procedures, result interpretation, and follow-up (14–17). In our questionnaire, Q2 and Q3 may have produced lower ratings partly because “common types” and “common symptoms” are ambiguous for rare, heterogeneous conditions and do not identify the locally screened panel. Their scores therefore cannot be interpreted solely as lack of disease knowledge. Future questionnaires should replace these broad phrases with panel-specific items, such as which conditions are included locally and why many screened IMDs may be asymptomatic at birth; they should also separate confidence ratings from objectively scored knowledge and undergo cognitive interviewing and pilot testing. Treatment methods were the least understood topic, reinforcing the need for functional knowledge of why screening is performed, what a positive result means, and what actions follow an abnormal result.

Cross-national evidence suggests that the attitude-understanding gap is recurrent but not demonstrably universal. In Thailand, a 2023 study of 717 pregnant women or partners and parents classified only 10% as having good objective knowledge while reporting positive attitudes toward current and expanded screening (26). In Türkiye, a 2023 survey of 407 postpartum mothers found generally inadequate knowledge; 52.8% had received newborn-screening information, most often from health professionals (27). These closer-income comparators broadly align with the coexistence of acceptance and limited understanding observed here. However, differences in populations, instruments, screening panels, timing, consent models, and service access prevent direct prevalence comparison and suggest that the magnitude and drivers of the gap are context-specific.

Q14 asked about personal initiative in seeking and reading IMD information, but the survey did not ask why women did or did not engage. Time constraints, restricted access to trustworthy and comprehensible information, uncertainty about source credibility, limited professional prompting, digital or health-literacy demands, and language barriers are plausible explanations, but none was measured and they should not be inferred from the Q14 score. Future questionnaires should separate information seeking from reading and use, include explicit barrier and preferred-channel items, and be supplemented by qualitative interviews.

The moderate association between perceived understanding and information engagement supports integrating newborn-screening education into routine antenatal contact rather than relying only on self-initiated searches. Maternal and child health nurses are well positioned to deliver a brief, staged explanation during antenatal visits, reinforce it with plain-language printed and low-bandwidth digital materials, provide a reliable route for questions, and use teach-back to confirm understanding. The 2025 ISNS guideline similarly places culturally appropriate information—ideally provided during pregnancy—within the screening pathway (13). A structured and accessible antenatal approach may therefore improve preparedness before families encounter time-sensitive sampling, recall, or confirmatory testing.

### 4.3 Favorable attitudes, intentions, and preparedness for follow-up

The high attitude and intention scores show that most participants endorsed newborn screening, health education, treatment, and family support. The strong attitude-intention correlation indicates that positive beliefs about the value of screening were closely connected with stated readiness to participate in screening and follow-up. This is compatible with evidence that parents generally value newborn screening and often support its continuation or expansion (17). It also provides an important service foundation: education can build on existing acceptance rather than beginning with resistance to screening.

Stated intentions should be translated into practical preparedness. Because this study measured pre-birth statements rather than postpartum behavior, it cannot determine whether the reported intentions were enacted, particularly when families must respond quickly to an unexpected screen-positive result. Antenatal counseling can bridge this gap by explaining that a positive screen is not a diagnosis, identifying who will contact the family, and clarifying the steps for repeat sampling, confirmatory testing, referral, and treatment. This preparation is clinically meaningful because false-positive results may generate considerable distress (18, 19), while a confirmed IMD can create sustained psychosocial and financial demands for families (20).

### 4.4 Sociodemographic patterns and implications for maternal and child health services

Although 15 demographic contrasts reached nominal significance, none remained significant after FDR correction, and adjusted R^2^ values of 0.010–0.039 left more than 96% of score variance unexplained. These models therefore provide no useful basis for identifying demographic subgroups or explaining domain scores, and the nominal contrasts should not be interpreted as evidence that a particular group has lower educational needs. Conversely, the low R^2^ values do not prove that subgroup inequities are absent; potentially relevant determinants such as the timing and quality of counseling, access to trustworthy information, health literacy, clinician recommendation, local panel availability, and cost were not measured. A universal antenatal education offer is supported primarily by the overall domain distributions, with individualized support according to expressed questions and service access, rather than by the regression models.

A concise nursing-led education module could cover the locally available core and optional panels; any additional payment; the timing and method of bloodspot collection; the non-diagnostic meaning of a positive screen; the recall and confirmatory-testing process; and routes to treatment, genetic counseling, and psychosocial support. Information should be delivered in plain language through both face-to-face and digital formats, with a reliable contact route for urgent recall questions. In this way, favorable attitudes can be converted into informed readiness, while the topics with the lowest perceived understanding receive focused attention.

### 4.5 Strengths and limitations

The study’s strengths include a large analytic sample, complete item data, preservation and transparent reporting of all 21 questionnaire responses, item-level presentation, examination of the four-domain internal structure, and full multivariable reporting with HC3 standard errors and FDR correction. The analysis also links domain findings to specific points in the antenatal-to-postnatal screening pathway, increasing their relevance for maternal and child health services.

The limitations should nevertheless be considered together. Participants were recruited from one tertiary hospital through purposive sampling and were predominantly urban and relatively well educated, which limits generalizability. In particular, the broad wording of Q2 and Q3 may have influenced perceived-understanding ratings, while Q14 did not capture time, access, credibility, literacy, language, or other barriers to information engagement. Adjusted R^2^ values of 0.010–0.039 and the absence of FDR-significant predictors show that the measured covariates explain little variation and cannot identify the determinants of the domain scores. The study did not observe postpartum screening, recall, diagnostic confirmation, treatment, or long-term care and did not measure cost, reimbursement, panel availability, clinician recommendation, or travel burden.

Future research should use cognitive interviews, pilot testing, and external validation to refine panel-specific Q2/Q3 content and barrier-sensitive Q14 content; distinguish objectively scored knowledge from self-perceived understanding; and measure information access, health literacy, professional communication, and service-level factors. Multicenter studies should include rural and lower-level facilities, use harmonized measures for comparison with similar-income settings, and follow women from pregnancy through newborn sampling, result communication, recall, confirmation, and treatment. Such work could determine whether structured antenatal education improves observed participation, preparedness, and continuity of follow-up.

## 5 Conclusion

### 5.1 Key findings

In this tertiary-hospital sample, pregnant women reported limited self-perceived understanding, favorable attitudes, moderate antenatal information engagement, and strong screening- and follow-up-related intentions. Perceived understanding was associated with engagement and attitudes with intentions, whereas understanding and attitudes were essentially unrelated. Sociodemographic and reproductive-history models explained only 1.0–3.9% of score variance and had no FDR-significant predictors.

### 5.2 Practical recommendations

A universal, nursing-led antenatal communication offer is recommended rather than demographic targeting. It should explain locally available panels, bloodspot collection, the non-diagnostic meaning of a positive result, recall, confirmation, treatment, and family support; use plain language, accessible printed and digital formats, teach-back, and a reliable contact route; and respond to individual information-access barriers.

### 5.3 Future research

Future studies should refine ambiguous Q2/Q3 wording and barrier-limited Q14 content through cognitive interviews and pilot testing, distinguish objective knowledge from perceived understanding, compare harmonized measures across similar-income settings, and prospectively test whether structured education improves actual screening participation, recall, and follow-up.

## Data Availability

The raw data supporting the conclusions of this article will be made available by the authors, without undue reservation.
