# Supplementary material for: Pregnant women’s perceived understanding and antenatal information needs regarding newborn screening for inherited metabolic diseases in southwestern China: a cross-sectional study
Source: Front Public Health. 2026 Oct 6;14:1865107. doi: 10.3389/fpubh.2026.1865107 (PMC13682302; doi:10.3389/fpubh.2026.1865107)
Supplement: Supplementary file 1 [file Table_1.docx]

## Supplementary materials

Supplementary Table S1. Exact wording and descriptive results for all 21 items

| **Item** | **Analytic domain** | **Exact wording** | **Mean (SD)** | **Score 4–5, n (%)** |
| --- | --- | --- | --- | --- |
| Q1 | Perceived understanding | I understand the basic concepts of newborn inherited metabolic diseases. | 2.46 (0.87) | 144 (10.9%) |
| Q2 |  | I understand the common types of newborn inherited metabolic diseases. | 2.25 (0.81) | 77 (5.8%) |
| Q3 |  | I understand the common symptoms of newborn inherited metabolic diseases. | 2.17 (0.78) | 57 (4.3%) |
| Q4 |  | I understand the diagnostic methods for newborn inherited metabolic diseases. | 2.14 (0.83) | 87 (6.6%) |
| Q5 |  | I understand the treatment methods for newborn inherited metabolic diseases. | 1.86 (0.65) | 12 (0.9%) |
| Q6 |  | I understand the preventive measures for newborn inherited metabolic diseases. | 2.07 (0.77) | 40 (3.0%) |
| Q7 |  | I understand the risk factors for newborn inherited metabolic diseases. | 2.19 (0.83) | 69 (5.2%) |
| Q8 | Attitudes | I think it is important to understand newborn inherited metabolic diseases for prevention and early treatment. | 4.27 (0.73) | 1,222 (92.6%) |
| Q9 |  | I believe genetic counseling plays an important role in preventing newborn inherited metabolic diseases. | 4.20 (0.71) | 1,201 (91.0%) |
| Q10 |  | I am willing to participate in health education activities regarding newborn inherited metabolic diseases. | 4.11 (0.70) | 1,161 (88.0%) |
| Q11 |  | I think regular screening for newborn inherited metabolic diseases is necessary. | 4.25 (0.70) | 1,219 (92.3%) |
| Q12 |  | I have a positive attitude toward the treatment of newborn inherited metabolic diseases. | 4.27 (0.69) | 1,235 (93.6%) |
| Q13 |  | I believe everyone has a responsibility to increase awareness of newborn inherited metabolic diseases. | 4.32 (0.69) | 1,247 (94.5%) |
| Q14 | Antenatal information engagement | I regularly seek and read information about newborn inherited metabolic diseases. | 3.54 (0.81) | 802 (60.8%) |
| Q15 |  | I have participated in health education activities regarding newborn inherited metabolic diseases. | 2.69 (1.02) | 359 (27.2%) |
| Q16 |  | I started learning about and preventing newborn inherited metabolic diseases during my pregnancy. | 3.12 (1.00) | 578 (43.8%) |
| Q17 | Screening and follow-up intentions | I regularly screen for newborn inherited metabolic diseases. | 3.97 (0.77) | 1,067 (80.8%) |
| Q18 |  | I actively seek advice from doctors and professionals to understand and prevent newborn inherited metabolic diseases. | 4.01 (0.75) | 1,099 (83.3%) |
| Q19 |  | I share and communicate knowledge about newborn inherited metabolic diseases with family and friends. | 3.94 (0.76) | 1,064 (80.6%) |
| Q20 |  | I encourage and support family and friends to undergo screening and treatment for newborn inherited metabolic diseases. | 4.03 (0.70) | 1,119 (84.8%) |
| Q21 |  | I follow the doctor’s advice for the treatment and care of newborn inherited metabolic diseases. | 4.14 (0.69) | 1,186 (89.8%) |

Supplementary Table S2. Full multivariable regression coefficients

| **Outcome** | **Predictor/contrast** | **B** | **HC3 SE** | **95% CI** | **p** | **FDR q** | **Adjusted R²** |
| --- | --- | --- | --- | --- | --- | --- | --- |
| Perceived understanding | Age 26–30 (vs 18–25) | −1.313 | 0.435 | −2.165 to −0.461 | 0.003 | 0.054 | 0.010 |
|  | Age 31–35 (vs 18–25) | −1.051 | 0.448 | −1.929 to −0.172 | 0.019 | 0.160 | 0.010 |
|  | Age 36–40 (vs 18–25) | −0.772 | 0.489 | −1.731 to 0.188 | 0.115 | 0.364 | 0.010 |
|  | Age ≥41 (vs 18–25) | −0.609 | 0.675 | −1.934 to 0.716 | 0.368 | 0.604 | 0.010 |
|  | Town (vs urban) | 1.123 | 0.352 | 0.433 to 1.814 | 0.001 | 0.051 | 0.010 |
|  | Rural (vs urban) | 0.722 | 0.501 | −0.260 to 1.705 | 0.149 | 0.370 | 0.010 |
|  | Associate degree (vs high school or below) | −0.070 | 0.345 | −0.748 to 0.607 | 0.838 | 0.929 | 0.010 |
|  | Bachelor’s degree (vs high school or below) | 0.490 | 0.369 | −0.233 to 1.213 | 0.184 | 0.407 | 0.010 |
|  | Master’s degree or above (vs high school or below) | 0.252 | 0.593 | −0.911 to 1.415 | 0.671 | 0.878 | 0.010 |
|  | Income 4,001–8,000 (vs ≤4,000 CNY) | 0.851 | 0.426 | 0.016 to 1.686 | 0.046 | 0.272 | 0.010 |
|  | Income 8,001–12,000 (vs ≤4,000 CNY) | 0.936 | 0.475 | 0.005 to 1.868 | 0.049 | 0.272 | 0.010 |
|  | Income 12,001–16,000 (vs ≤4,000 CNY) | 1.257 | 0.547 | 0.184 to 2.331 | 0.022 | 0.167 | 0.010 |
|  | Income 16,001–20,000 (vs ≤4,000 CNY) | 1.106 | 0.580 | −0.032 to 2.244 | 0.057 | 0.272 | 0.010 |
|  | Income ≥20,001 (vs ≤4,000 CNY) | 1.108 | 0.593 | −0.056 to 2.272 | 0.062 | 0.272 | 0.010 |
|  | Commercial insurance: yes | 0.194 | 0.281 | −0.357 to 0.745 | 0.490 | 0.740 | 0.010 |
|  | Family history of genetic disease: yes | −0.340 | 0.824 | −1.956 to 1.276 | 0.680 | 0.878 | 0.010 |
|  | History of miscarriage: yes | −0.187 | 0.332 | −0.840 to 0.465 | 0.573 | 0.837 | 0.010 |
|  | Diagnosed genetic disease: yes | 0.332 | 0.933 | −1.500 to 2.163 | 0.722 | 0.880 | 0.010 |
|  | Diagnosed genetic variant: yes | 0.370 | 0.527 | −0.663 to 1.403 | 0.482 | 0.740 | 0.010 |
|  | Diagnosed chromosomal abnormality: yes | −0.876 | 0.672 | −2.194 to 0.442 | 0.193 | 0.407 | 0.010 |
|  | Previous child with IMD: yes | 2.568 | 0.993 | 0.619 to 4.517 | 0.010 | 0.113 | 0.010 |
|  | Ethnicity: minority | −0.171 | 0.597 | −1.342 to 0.999 | 0.774 | 0.890 | 0.010 |
| Attitudes | Age 26–30 (vs 18–25) | 0.503 | 0.349 | −0.181 to 1.188 | 0.149 | 0.370 | 0.034 |
|  | Age 31–35 (vs 18–25) | −0.090 | 0.366 | −0.809 to 0.629 | 0.806 | 0.904 | 0.034 |
|  | Age 36–40 (vs 18–25) | −0.359 | 0.387 | −1.118 to 0.400 | 0.353 | 0.591 | 0.034 |
|  | Age ≥41 (vs 18–25) | −0.205 | 0.625 | −1.431 to 1.021 | 0.743 | 0.887 | 0.034 |
|  | Town (vs urban) | −0.481 | 0.273 | −1.016 to 0.055 | 0.079 | 0.296 | 0.034 |
|  | Rural (vs urban) | −1.105 | 0.413 | −1.916 to −0.294 | 0.008 | 0.113 | 0.034 |
|  | Associate degree (vs high school or below) | 0.487 | 0.292 | −0.086 to 1.060 | 0.096 | 0.333 | 0.034 |
|  | Bachelor’s degree (vs high school or below) | 0.891 | 0.299 | 0.304 to 1.477 | 0.003 | 0.054 | 0.034 |
|  | Master’s degree or above (vs high school or below) | 0.774 | 0.541 | −0.287 to 1.835 | 0.153 | 0.370 | 0.034 |
|  | Income 4,001–8,000 (vs ≤4,000 CNY) | 0.088 | 0.344 | −0.587 to 0.764 | 0.798 | 0.904 | 0.034 |
|  | Income 8,001–12,000 (vs ≤4,000 CNY) | 0.656 | 0.374 | −0.078 to 1.390 | 0.080 | 0.296 | 0.034 |
|  | Income 12,001–16,000 (vs ≤4,000 CNY) | 0.623 | 0.429 | −0.218 to 1.464 | 0.146 | 0.370 | 0.034 |
|  | Income 16,001–20,000 (vs ≤4,000 CNY) | 0.030 | 0.497 | −0.945 to 1.004 | 0.952 | 0.965 | 0.034 |
|  | Income ≥20,001 (vs ≤4,000 CNY) | 0.608 | 0.479 | −0.332 to 1.549 | 0.205 | 0.419 | 0.034 |
|  | Commercial insurance: yes | 0.410 | 0.222 | −0.026 to 0.845 | 0.065 | 0.272 | 0.034 |
|  | Family history of genetic disease: yes | −0.206 | 0.476 | −1.140 to 0.727 | 0.665 | 0.878 | 0.034 |
|  | History of miscarriage: yes | −0.124 | 0.308 | −0.728 to 0.480 | 0.687 | 0.878 | 0.034 |
|  | Diagnosed genetic disease: yes | 0.986 | 0.601 | −0.193 to 2.164 | 0.101 | 0.333 | 0.034 |
|  | Diagnosed genetic variant: yes | 0.479 | 0.402 | −0.310 to 1.268 | 0.234 | 0.453 | 0.034 |
|  | Diagnosed chromosomal abnormality: yes | −0.756 | 0.520 | −1.777 to 0.265 | 0.147 | 0.370 | 0.034 |
|  | Previous child with IMD: yes | −0.054 | 1.224 | −2.455 to 2.347 | 0.965 | 0.965 | 0.034 |
|  | Ethnicity: minority | 0.119 | 0.405 | −0.676 to 0.914 | 0.769 | 0.890 | 0.034 |
| Antenatal information engagement | Age 26–30 (vs 18–25) | −0.303 | 0.225 | −0.744 to 0.138 | 0.178 | 0.407 | 0.039 |
|  | Age 31–35 (vs 18–25) | −0.242 | 0.228 | −0.688 to 0.205 | 0.288 | 0.510 | 0.039 |
|  | Age 36–40 (vs 18–25) | −0.090 | 0.247 | −0.575 to 0.394 | 0.714 | 0.880 | 0.039 |
|  | Age ≥41 (vs 18–25) | 0.135 | 0.387 | −0.625 to 0.895 | 0.727 | 0.880 | 0.039 |
|  | Town (vs urban) | 0.335 | 0.178 | −0.014 to 0.685 | 0.060 | 0.272 | 0.039 |
|  | Rural (vs urban) | 0.037 | 0.246 | −0.446 to 0.521 | 0.879 | 0.952 | 0.039 |
|  | Associate degree (vs high school or below) | −0.255 | 0.176 | −0.601 to 0.091 | 0.148 | 0.370 | 0.039 |
|  | Bachelor’s degree (vs high school or below) | −0.604 | 0.189 | −0.975 to −0.232 | 0.001 | 0.051 | 0.039 |
|  | Master’s degree or above (vs high school or below) | −0.950 | 0.301 | −1.541 to −0.359 | 0.002 | 0.051 | 0.039 |
|  | Income 4,001–8,000 (vs ≤4,000 CNY) | −0.414 | 0.224 | −0.852 to 0.025 | 0.065 | 0.272 | 0.039 |
|  | Income 8,001–12,000 (vs ≤4,000 CNY) | −0.270 | 0.245 | −0.751 to 0.212 | 0.272 | 0.500 | 0.039 |
|  | Income 12,001–16,000 (vs ≤4,000 CNY) | −0.715 | 0.282 | −1.268 to −0.162 | 0.011 | 0.116 | 0.039 |
|  | Income 16,001–20,000 (vs ≤4,000 CNY) | −0.761 | 0.312 | −1.373 to −0.149 | 0.015 | 0.136 | 0.039 |
|  | Income ≥20,001 (vs ≤4,000 CNY) | −0.784 | 0.303 | −1.377 to −0.190 | 0.010 | 0.113 | 0.039 |
|  | Commercial insurance: yes | 0.260 | 0.138 | −0.010 to 0.530 | 0.059 | 0.272 | 0.039 |
|  | Family history of genetic disease: yes | 0.250 | 0.352 | −0.442 to 0.941 | 0.479 | 0.740 | 0.039 |
|  | History of miscarriage: yes | −0.321 | 0.173 | −0.661 to 0.019 | 0.064 | 0.272 | 0.039 |
|  | Diagnosed genetic disease: yes | −0.276 | 0.433 | −1.125 to 0.574 | 0.525 | 0.779 | 0.039 |
|  | Diagnosed genetic variant: yes | 0.101 | 0.249 | −0.386 to 0.589 | 0.684 | 0.878 | 0.039 |
|  | Diagnosed chromosomal abnormality: yes | 0.161 | 0.336 | −0.498 to 0.820 | 0.632 | 0.878 | 0.039 |
|  | Previous child with IMD: yes | 0.727 | 0.416 | −0.088 to 1.543 | 0.081 | 0.296 | 0.039 |
|  | Ethnicity: minority | −0.106 | 0.347 | −0.788 to 0.575 | 0.759 | 0.890 | 0.039 |
| Screening and follow-up intentions | Age 26–30 (vs 18–25) | 0.018 | 0.298 | −0.567 to 0.604 | 0.951 | 0.965 | 0.010 |
|  | Age 31–35 (vs 18–25) | −0.352 | 0.298 | −0.937 to 0.233 | 0.238 | 0.453 | 0.010 |
|  | Age 36–40 (vs 18–25) | −0.475 | 0.322 | −1.106 to 0.157 | 0.140 | 0.370 | 0.010 |
|  | Age ≥41 (vs 18–25) | −0.190 | 0.500 | −1.171 to 0.792 | 0.704 | 0.880 | 0.010 |
|  | Town (vs urban) | −0.248 | 0.252 | −0.743 to 0.247 | 0.325 | 0.554 | 0.010 |
|  | Rural (vs urban) | −0.437 | 0.334 | −1.091 to 0.218 | 0.191 | 0.407 | 0.010 |
|  | Associate degree (vs high school or below) | −0.013 | 0.255 | −0.514 to 0.487 | 0.958 | 0.965 | 0.010 |
|  | Bachelor’s degree (vs high school or below) | 0.116 | 0.263 | −0.401 to 0.632 | 0.660 | 0.878 | 0.010 |
|  | Master’s degree or above (vs high school or below) | 0.203 | 0.423 | −0.627 to 1.033 | 0.632 | 0.878 | 0.010 |
|  | Income 4,001–8,000 (vs ≤4,000 CNY) | −0.034 | 0.308 | −0.639 to 0.570 | 0.911 | 0.965 | 0.010 |
|  | Income 8,001–12,000 (vs ≤4,000 CNY) | 0.393 | 0.335 | −0.265 to 1.051 | 0.241 | 0.453 | 0.010 |
|  | Income 12,001–16,000 (vs ≤4,000 CNY) | 0.374 | 0.375 | −0.361 to 1.110 | 0.318 | 0.553 | 0.010 |
|  | Income 16,001–20,000 (vs ≤4,000 CNY) | −0.037 | 0.426 | −0.872 to 0.799 | 0.931 | 0.965 | 0.010 |
|  | Income ≥20,001 (vs ≤4,000 CNY) | 0.660 | 0.402 | −0.130 to 1.449 | 0.101 | 0.333 | 0.010 |
|  | Commercial insurance: yes | 0.292 | 0.188 | −0.077 to 0.660 | 0.121 | 0.370 | 0.010 |
|  | Family history of genetic disease: yes | 0.071 | 0.449 | −0.809 to 0.951 | 0.874 | 0.952 | 0.010 |
|  | History of miscarriage: yes | −0.342 | 0.264 | −0.860 to 0.175 | 0.194 | 0.407 | 0.010 |
|  | Diagnosed genetic disease: yes | 0.608 | 0.572 | −0.515 to 1.731 | 0.288 | 0.510 | 0.010 |
|  | Diagnosed genetic variant: yes | 0.024 | 0.348 | −0.658 to 0.706 | 0.945 | 0.965 | 0.010 |
|  | Diagnosed chromosomal abnormality: yes | −0.237 | 0.467 | −1.154 to 0.680 | 0.613 | 0.878 | 0.010 |
|  | Previous child with IMD: yes | 1.499 | 0.737 | 0.054 to 2.944 | 0.042 | 0.272 | 0.010 |
|  | Ethnicity: minority | 0.557 | 0.389 | −0.207 to 1.321 | 0.153 | 0.370 | 0.010 |

*Reference groups: age 18–25 years; urban residence; high school or below; monthly household income ≤4,000 CNY; Han ethnicity; and “no” for binary variables. HC3 robust standard errors were used. FDR q values were calculated jointly across all non-intercept coefficients within this manuscript version.*
